# The smart activatable P2&3TT probe allows accurate, fast, and highly sensitive detection of *Staphylococcus aureus* in clinical blood culture samples

**DOI:** 10.1038/s41598-020-76254-4

**Published:** 2020-11-05

**Authors:** Marina López-Álvarez, Marjolein Heuker, Jorrit W. A. Schoenmakers, Gooitzen M. van Dam, James O. McNamara, Jan Maarten van Dijl, Marleen van Oosten

**Affiliations:** 1 Department of Medical Microbiology, University Medical Center Groningen, Hanzeplein 1, PO BOX 30001, 9700 RB Groningen, The Netherlands; 2 Department of Surgery, University Medical Center Groningen, Groningen, The Netherlands; 3 Department of Internal Medicine, Roy J. and Lucille A. Carver College of Medicine, University of Iowa, Iowa City, IA USA; 4 Nuclease Probe Technologies, Inc., Lowell, MA USA

**Keywords:** Microbiology techniques, Infection, Bacteria, Bacteriology, Diagnostic markers

## Abstract

*Staphylococcus aureus* bacteraemia (SAB) is associated with high mortality and morbidity rates. Yet, there is currently no adequate diagnostic test for early and rapid diagnosis of SAB. Therefore, this study was aimed at exploring the potential for clinical implementation of a nuclease-activatable fluorescent probe for early diagnosis of SAB. To this end, clinical blood culture samples from patients with bloodstream infections were incubated for 1 h with the “smart” activatable P2&3TT probe, the total assay time being less than 2 h. Cleavage of this probe by the secreted *S. aureus* enzyme micrococcal nuclease results in emission of a readily detectable fluorescence signal. Incubation of *S. aureus*-positive blood culture samples with the P2&3TT probe resulted in 50-fold higher fluorescence intensity levels than incubation with culture-negative samples. Moreover, incubation of the probe with non-*S. aureus*-positive blood cultures yielded essentially background fluorescence intensity levels for cultures with Gram-negative bacteria, and only ~ 3.5-fold increased fluorescence intensity levels over background for cultures with non-*S. aureus* Gram-positive bacteria. Importantly, the measured fluorescence intensities were dose-dependent, and a positive signal was clearly detectable for *S. aureus*-positive blood cultures with bacterial loads as low as ~ 7,000 colony-forming units/mL. Thus, the nuclease-activatable P2&3TT probe distinguishes clinical *S. aureus*-positive blood cultures from non-*S. aureus*-positive blood cultures and culture-negative blood, accurately, rapidly and with high sensitivity. We conclude that this probe may enhance the diagnosis of SAB.

## Introduction

Bloodstream infections (BSIs) are serious clinical conditions with high rates of morbidity and mortality^1–3^. *Staphylococcus aureus* is the leading cause of nosocomial bacteraemia and the second most frequent cause of community-acquired bacteraemia^4^. Despite the availability of appropriate antibiotics, annual mortality rates for *S. aureus* bacteraemia (SAB) remain high with ~ 2 to 10 deaths per 100,000 population^5,6^. Currently, the low bacterial loads in blood of bacteraemia patients delay accurate diagnosis of SAB and appropriate antimicrobial therapy^4,7^. About 18% of the patients with SAB have circulating bacteria at titres of less than 0.04 colony-forming units (CFU) per mL of blood^8^. In ~ 25% of the SAB cases, bacterial titres range between 1–10 CFU/mL, but they may reach over 100 CFU/mL in severe bacteraemia cases^7–9^.

The current diagnosis of bacteraemia relies on blood culture methods that take days. Therefore, initial treatment of patients with suspected bacteraemia is based on empiric infection management with broad-spectrum antibiotics, while traditional culture methods are performed to identify the causative organism^4^. As a consequence of the resulting use of broad-spectrum antibiotics, multi-drug-resistant bacteria are more likely to emerge, complicating treatment and leading to worse outcomes. Moreover, initial treatment can be wrong if a *S. aureus* infection is not suspected.

A highly promising molecular detection approach for rapid and culture-independent detection of *S. aureus* infections is based on enzymatic activity of micrococcal nuclease (MN), a protein also referred to as staphylococcal nuclease (SNase) or thermonuclease (TNase), which is specifically and invariably secreted by *S. aureus*^10–15^. The signal-amplifying ability of MN and its relative abundance provide a very sensitive means of detecting *S. aureus*, possibly enabling diagnosis of SAB in hours instead of days^4^.

The present study was aimed at validating the potential for clinical implementation of a nuclease-activatable fluorescent probe named P2&3TT for diagnosis of SAB. Of note, in addition to MN, *S. aureus* produces a second nuclease (Nuc2), a surface-bound nuclease that exhibits substantially less activity than MN^13,16,17^. Upon specific cleavage of this probe by the secreted MN, it emits a readily detectable fluorescence signal^12,18^. Here we report that the P2&3TT probe can distinguish clinical *S. aureus*-positive blood cultures from clinical non-*S. aureus*-positive blood cultures and culture-negative blood, rapidly and with high sensitivity.

## Results

### Evaluation of the P2&3TT probe in blood culture samples

**Figure 1:**
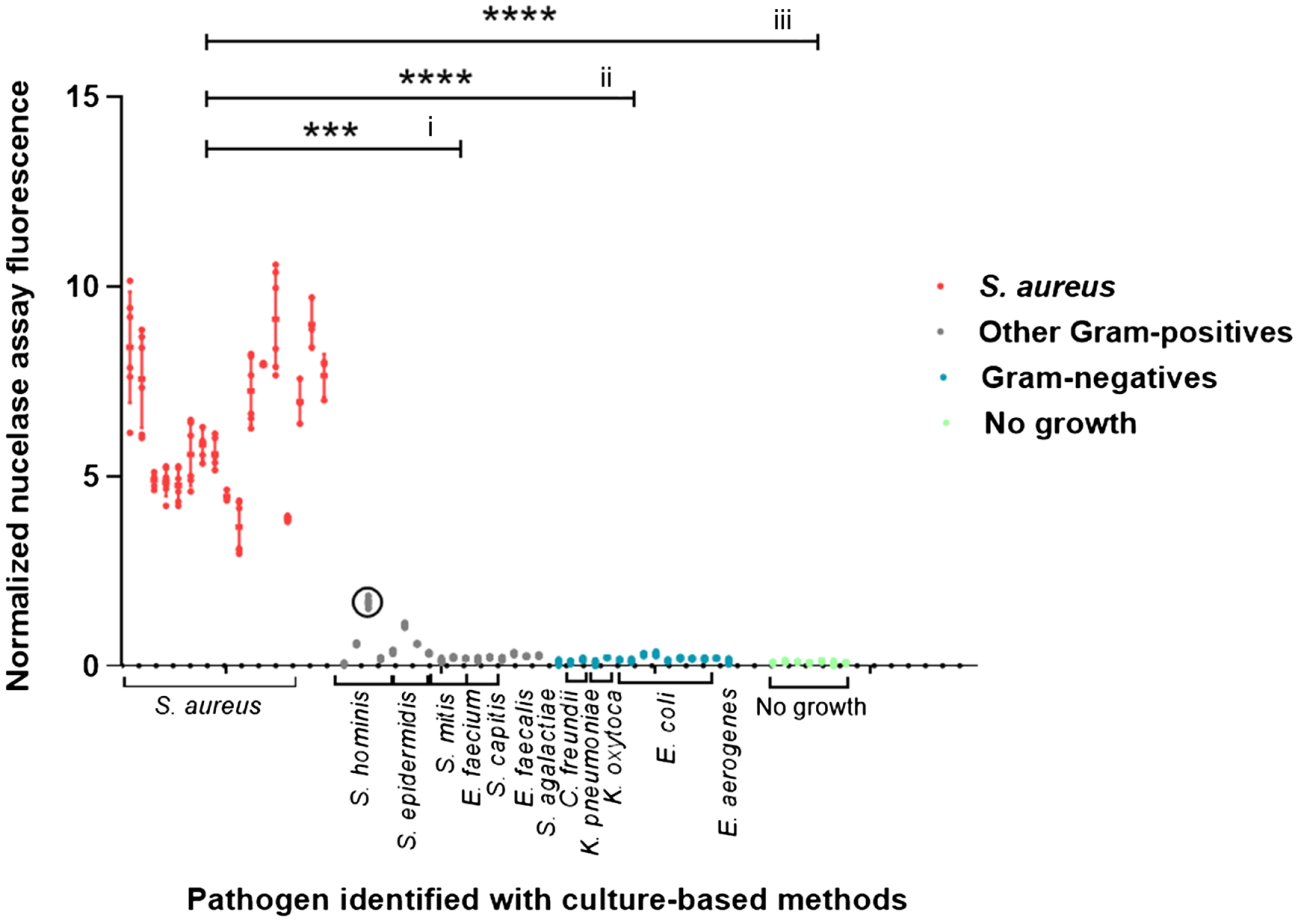
Evaluation of the P2&3TT probe in blood cultures. Nuclease activity assays were carried out with blood culture samples from: patients with negative-culture outcomes (n = 7, green), and patients with positive blood culture outcomes for non-*S. aureus* pathogens including both Gram-positive bacteria (n = 17, grey) and Gram-negative bacteria (n = 15, blue), or *S. aureus* (n = 17, red) (probe concentration 3.9 µM). The detected pathogens are indicated. After correction for the background based on subtraction of the fluorescence value of each blood sample without the probe, fluorescence values were normalized to the average fluorescence values of the probe signal in buffer. Variations in nuclease activity in the different blood samples are indicated by dots. Of note, a *S. hominis-*infected blood culture co-infected with *S. saprophyticus* and *S. epidermidis* is marked with a circle. The fluorescence intensity of this sample (1.67, SD 0.16) is higher than expected based on the T/B value for *S. hominis.* Possibly, this sample contained also a minor undetected fraction of *S. aureus*, or the detected *S. hominis*, *S. epidermidis* or *S. saprophyticus* secreted a nuclease that can cleave the probe. To test statistical significance, the nuclease activity measured in *S. aureus*-positive blood culture samples was compared with that in blood cultures testing positive for Gram-positive non-*S. aureus* bacteria (i), Gram-negative bacteria (ii), or negative blood culture samples (iii). **** Indicates P < 0.0001, *** indicates P = 0.0006 (Kruskal–Wallis GraphPad Prism 8.0.1).

To test whether the P2&3TT probe allows the direct detection of *S. aureus* in blood cultures of SAB patients, 17 blood cultures, identified as *S. aureus*-positive with conventional culture-based methods, were spiked with calcium chloride, heat-treated and centrifuged. Notably, the heat treatment was previously shown to increase the detection sensitivity of MN by several orders of magnitude through the inactivation of potential MN inhibitory antibodies in human serum^4^. The supernatants were then incubated with the probe at a concentration of 3.9 µM. Indeed, probe activation was observed in all 17 cases as shown in Fig. 1. In contrast, incubation of the probe with culture-negative blood (n = 7) or non-*S. aureus-*positive blood cultures (n = 32) did not result in probe activation and, consequently, only marginal fluorescence was detected. The differences in fluorescence signals obtained upon probe incubation with *S. aureus-*positive blood cultures compared to *S. aureus*-negative blood cultures containing other Gram-positive bacteria (p = 0.0006) or Gram-negative bacteria (p < 0.0001), or compared to culture-negative blood (p < 0.0001) were statistically significant (Fig. 1).

**Figure 2:**
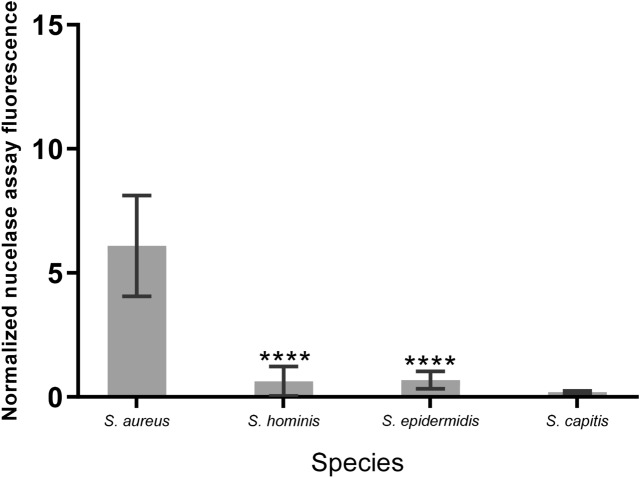
Nuclease activity in blood cultures of *Staphylococcus* species. Average nuclease activity in blood cultures of *S. aureus*, *S. hominis*, *S. epidermidis* and *S. capitis.* 10 µl of blood culture supernatant was incubated with the P2&3TT probe for 1 h at 37˚C and fluorescence was measured and corrected as previously indicated. **** Indicates P < 0.0001. (Brown-Forsythe and Welch GraphPad Prism 8.0.1).

To calculate T/B ratios, for each investigated blood culture sample the background fluorescence of the sample without probe was subtracted from the fluorescence measured for the sample incubated with probe. Next, the resulting background-corrected fluorescence value of the sample was normalized by dividing it by the signal of the P2&3TT probe in buffer. This showed that probe incubation in *S. aureus-*positive blood culture samples resulted in an average normalized fluorescence intensity of 6.08 (SD 2.03), whereas this number was 0.12 (SD 0.09) for culture-negative blood culture samples. Probe incubation in non-*S. aureus*-positive blood cultures yielded an average normalized fluorescence intensity of 0.17 (SD 0.08) for Gram-negative bacteria and 0.42 (SD 0.41) for non-staphylococcal Gram-positive bacteria. Probe incubation with blood cultures positive for other *Staphylococcus* species resulted in average normalized fluorescence intensities of 0.63 (SD 0.59) for *Staphylococcus hominis*, 0.67 (SD 0.34) for *Staphylococcus epidermidis*, and 0.19 (SD 0.04) for *Staphylococcus capitis* (Fig. 2).

### Evaluation of P2&3TT probe sensitivity for *S. aureus* detection in blood

**Figure 3:**
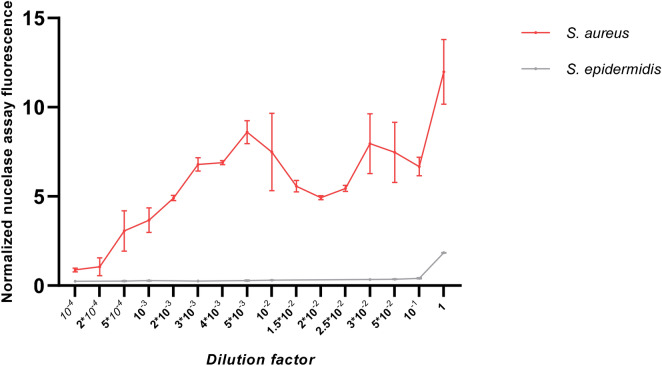
Evaluation of the P2&3TT probe in blood cultures diluted with blood from healthy volunteers. Nuclease activity assays were carried out with dilutions from *S. aureus and S. epidermidis*-positive blood cultures (Fig. 1) (probe concentration 3.9 µM). The initial amount of bacteria in the blood cultures (corresponding to dilution factor 1) was 7.1 × 10^7^ CFU/mL and 2.16 × 10^9^ CFU/mL for *S. aureus* and *S. epidermidis* respectively. After the correction for the background based on the subtraction of the fluorescence value of each blood sample without the probe, fluorescence values were normalized to the average fluorescence values of the probe signal in buffer.

Considering the strong performance of the probe in blood cultures, we next evaluated its potential in detecting *S. aureus* directly in patient blood samples. An *S. aureus*-positive blood culture was step-wise diluted in healthy donor blood to simulate *S. aureus*-positive, uncultured blood. We spiked the sample with calcium chloride, heated and incubated the heat-treated sample supernatants with 3.9 µM probe. CFU counting was performed in parallel to determine the input bacterial loads. As expected, the highest fluorescence intensity was observed with undiluted *S. aureus-*positive blood culture, but with as much as 10^4^-fold dilution corresponding to ~ 7000 CFU/mL, a fluorescence signal that was clearly distinguishable from the control signal of the corresponding *S. epidermidis-*positive blood culture dilution was obtained (Fig. 3).

**Figure 4:**
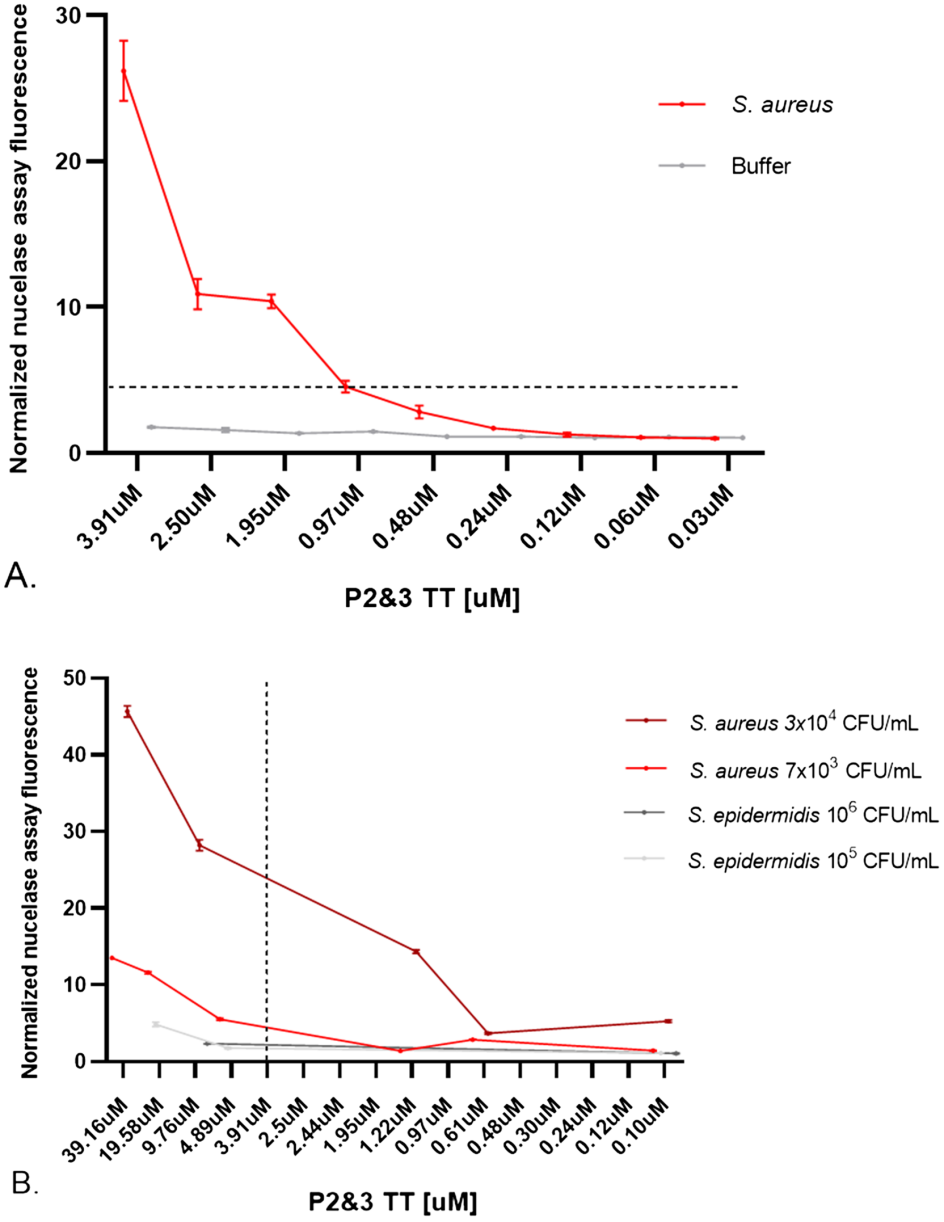
Sensitivity of the P2&3TT probe. The P2&3TT probe was diluted in buffer and incubated at 37˚C for 1 h (**A**) in blood cultures from SAB patients, or (**B**) in blood cultures diluted with blood from healthy volunteers. Subsequently, fluorescence was measured. Error bars indicate standard deviations of triplicate measurements. The horizontal line represents fluorescence signal considered as positive for *S. aureus* based on the results presented in Fig. 1. The vertical line represents the probe concentration selected as optimal for SAB blood culture. Normalization of the fluorescence measurements was done by dividing each fluorescent measurement by the signal of each blood sample without the P2&3TT probe.

Lastly, to determine whether *S. aureus*-positive blood could be identified with lower concentrations of P2&3TT probe, we incubated two SAB blood culture samples with step-wise reduced P2&3TT probe concentrations. This resulted in step-wise reduction of the fluorescence signal, which was still clearly detectable at probe concentrations of ~ 0.5 µM (Fig. 4A). In parallel, we investigated whether a substantially lowered *S. aureus* load would be detectable with the P2&3TT probe at different concentrations. *S. aureus*-positive cultured blood was 10^4^-fold diluted with blood from healthy volunteers and incubated with different concentrations of probe. For the 10^4^-fold diluted *S. aureus*-positive blood sample, the probe used at the standard concentration of 3.9 µM still gave a clear fluorescence signal that could be distinguished from that of the *S. epidermidis* control. However, this signal was strongly enhanced by using higher probe concentrations, which was tested for a maximum final probe concentration of 39.1 µM (Fig. 4B). From these sensitivity studies which, together, involved three different *S. aureus*-positive blood cultures with triplicate measurements for each of the three biological replicates (Figs. 3 and 4), we conclude that the P2&3TT probe can enable rapid and precise diagnosis of SAB.

## Discussion

Considering the high risk of BSIs for patients, a point-of-care test that provides reliable diagnoses within minutes would be ideal. Unfortunately, current diagnostic methods take much longer, ranging between one to seven days, or more. This delayed diagnosis results in poorer outcome for patients. The empirical broad-spectrum antimicrobial therapy initiated when BSI is suspected can exhibit side-effects and is prone to elicit antibiotic resistance^19^. In worst case, empirical antimicrobial therapy does not cover the causative micro-organism, potentially leading to death of the patient. These concerns are particularly relevant for SAB, because of the high *S. aureus* virulence^18,20^. Since the previously developed P2&3TT probe may offer significantly faster SAB detection^18^, this validation study aimed at assessing its sensitivity and specificity using clinical blood culture samples.

Here we demonstrate recovery of active MN from *S. aureus*-positive blood cultures. We specifically detect this enzyme with the P2&3TT probe, even after 10^4^-fold dilution of cultures prior to probe incubation (corresponding to ~ 7,000 CFU/mL). Accordingly, we conclude that the detection of SAB with the P2&3TT probe is at least 10^4^-fold more sensitive than the sensitivity achieved by diagnostic culturing based on the detection of CO_2_ as implemented in the BD BACTEC blood culture system (BACTEC Fluorescent Series User’s Manual)^16^. Of note, this will generally apply to SAB since the P2&3TT probe, under presently applied assay conditions, is unlikely to detect BSIs caused by organisms other than *S. aureus*. Further, the nuclease assay takes less than 2 h and is inexpensive. Methods currently in common use require plating on agar media and incubating until colonies are formed, taking a minimum of 12–14 hours^1^.

Our data support the notion that the P2&3TT probe assay can provide the needed specificity for rapid *S. aureus* identification. However, we cannot exclude the possibility that rare infections (e.g. caused by uncommon coagulase-negative staphylococci such as *S. argenteus*, *S. hyicus*, *S. intermedius*, and *S. schweitzeri*) may yield false positive results since they were not encountered in our proof-of-concept study^21–23^. Based on its time-to-result, P2&3TT probe-based detection of *S. aureus* provides the means to expedite identification of *S. aureus* bactaeremia by at least several hours versus current methods in common use. As a standalone assay that is specific for a single bloodstream pathogen, this assay will not replace current methods which detect pathogens regardless of their species. Rather, we envision its use as a complementary tool to current methods (e.g. mass spectrometry- and PCR-based identification of culture isolates) that provides more rapid identification of the most impactful bacterial bloodstream pathogen.

Lastly, in the present experimental set-up, we have sampled blood culture bottles which, for ethical reasons, was done once culture-positive signals were obtained, or when blood samples tested negative after 4–7 days of culturing. Since CO_2_ production during blood culturing is detected with fluorescence tracers in some blood culture devices, it is well conceivable that next-generation MN-activatable probes can be directly implemented in diagnostic blood culture systems. We conclude that MN-activatable probes, like the P2&3TT probe can enable rapid and precise diagnosis of SAB.

## Methods

### Nuclease-activatable P2&3TT probe

The structure of the nuclease-activatable P2&3TT probe was previously described^4,18^. In short, it consists of an oligonucleotide comprising 2′- O-methyl modified uridines flanking a pair of unmodified deoxythymidines, which is coupled to fluorescein amidite on the 5′-end and the ZEN and Iowa Black RQ quenchers on the 3′-end. For the present study, it was synthesized and purified by Integrated DNA Technologies, Inc. (IDT; Coralville, IA). The lyophilized probe was dissolved in 10 mM Tris–HCl pH 8.0, 1 mM EDTA to a final concentration of 391.6 μM and stored at -80 °C. For the assay, 1 µL of probe was diluted in 9 µL of 10 mM Tris–HCl pH 9.0, 10 mM CaCl_2_ to yield a working stock of 39.16 µM^18^.

### Blood culture samples

Clinical blood cultures generated in BD Bactec bottles (Becton, Dickinson and Company, USA) were collected from the department of Medical Microbiology (University Medical Center Groningen [UMCG]) after one to two weeks of culturing and storage. All blood culture samples were incubated until marked positive by a BD BACTEC FX Blood Culture System, or until unloaded as culture-negative after 4–7 days. Each blood sample was sub-cultured in parallel according to standard procedures to verify the microbiological diagnosis by plating and MALDI-TOF mass spectrometry.

### Blood samples from healthy volunteers

Blood from healthy volunteers was collected in BD Vacutainer Heparin tubes to perform sensitivity assays on the day of the experiment. These blood samples were also used to prepare dilutions of infected-blood cultures to assess the sensitivity of the P2&3TT probe.

### Determination of bacterial titres

To determine CFUs, serially diluted blood cultures were plated on blood agar media immediately prior to carrying out the nuclease assays, and the respective plates were incubated overnight at 37 °C.

### Detection of micrococcal nuclease activity in blood

Blood culture samples prior or after dilution with blood from healthy volunteers were supplemented with CaCl_2_ (10 µM), heated to 90 °C for 20 min, and centrifuged for 10 min (16,000 rpm, room temperature). 10µL of the supernatant were incubated with the P2&3TT probe for 1 h at 37 °C^4^. Then, 290 µl of the stop solution (10 mM Tris–HCl pH 9.0, 10 mM EDTA) were added to stop activity of the micrococcal nuclease. The stop solution works by restraining the free Ca^2+^ ions needed for activity of the nuclease. Afterwards, 95 µl aliquots of each stopped reaction sample were transferred in triplicate to a flat-bottomed transparent 96-well plate and fluorescence was measured in a Biotek Synergy 2.0 plate reader (BioTek Instruments, Inc., USA). Fluorescence was measured with filters optimal for FITC; excitation was set at 480 nm and emission at 520 nm, with optics position at “bottom”. All measurements were carried out in triplicate at 37 °C without shaking.

### Statistical analyses

In each experiment, unless stated otherwise, mean fluorescence levels of background controls (e.g., blood without the P2&3TT probe) were subtracted from the signal of the measured condition and divided by the signal of the P2&3TT probe in buffer to calculate a target-to-background (T/B) signal. The blood was divided into four equal volumes, 1 mL each, of which two were incubated with the probe and two were incubated in parallel without probe. All fluorescence measurements were made in triplicate. All p-values were calculated using a one-way ANOVA in GraphPad Prism 8.1.0. P-values < 0.05 were considered significant.

### Ethical approval

Permission for this study was obtained via the Medical Ethical Review Board Committee of the UMCG (permission number METc2017/098). The study was performed with adherence to the guidelines of the Declaration of Helsinki and local regulations, and all patient samples were treated pseudo-anonymously. Of note, during the present study period, patients admitted to the UMCG complied with hospital guidelines in an opt-out research consent procedure. Consequently, individual written consent was not required for inclusion of their diagnostic waste materials in our study, as consent was given through this opt-out practise unless stated otherwise in the medical file. Blood donations from healthy volunteers were collected with approval of the medical ethics committee of the UMCG (approval no. METc 2016/621). All blood donations were obtained after written informed consent from all volunteers, in accordance to the Helsinki Guidelines and local regulations, and all the samples were anonymized.

### Transparency declaration

J.O.M. is founder and CEO of Nuclease Probe Technologies Inc., and he holds patents on oligonucleotide-based nuclease-activatable probes for detection of bacterial nucleases. G.M.v.D. is CEO, founder and shareholder of AxelaRx / TRACER group, CSO AxelaRx Biosciences Inc. The other authors have no conflicts of interest to disclose.
